# Analytical formulation of spatiotemporal modulated graphene-based waveguides using Floquet-Bloch theory

**DOI:** 10.1038/s41598-024-56815-7

**Published:** 2024-03-27

**Authors:** Mahsa Valizadeh, Leila Yousefi, MirFaez Miri

**Affiliations:** 1 School of Electrical and Computer Engineering, College of Engineering, University of Tehran, Tehran, 1417614411 Iran; 2 Department of Physics, University of Tehran, Tehran, 14395547 Iran; 3 Engineering and Informatics School, University of Sussex, Falmer, BN1 9RH UK

**Keywords:** Electrical and electronic engineering, Terahertz optics, Nanophotonics and plasmonics

## Abstract

In this work, an analytical model to study graphene-based spatiotemporal modulated structures is developed and verified through comparison with full wave numerical simulations. Graphene is an ideal material for realizing spatiotemporal modulated structures at high frequencies of THz and optics. In this analysis, the electromagnetic response of studied structures is expressed in terms of weighted Floquet-Bloch modes supported by the structure, while graphene is modeled by a spatiotemporal modulated surface current that imposes certain boundary conditions on the modes. The developed analytical technique is a comprehensive tool and can be used for accurate modeling of different kinds of spatiotemporal devices including lossy, guided, and leaky wave structures. To demonstrate the accuracy of the model, two plasmonic waveguides with space and time modulated graphene conductivity are analyzed and their interband and intraband transition between modes are thoroughly investigated. Using the developed analytical model, spatiotemporal modulation phenomena such as mode conversion, wave amplification and nonreciprocal response are explored and discussed for the studied structures.

Spatiotemporal modulated structures with periodic variations in one or more of their electromagnetic properties (e.g., constitutive parameters) in both time and space have attracted much attention^1–11^. These structures exhibit interesting behaviors such as nonreciprocal response^1–9^, asymmetric band gap in the dispersion diagram^10^, frequency mixing^11^, and mode conversion^1,5^ which can be exploited for various applications. These unique features are not observed in static periodic structures^12–17^ or purely time-varying ones^18–21^. Thus spatiotemporal modulated structures are expected to replace their conventional counterparts which employ huge magnetic-biased components^22,23^ or nonlinear structures with high power requirements^24–28^.

So far, most of the spatiotemporal modulated structures have been studied and realized at microwave frequencies^2–4,6,8,10,11,29–32^. Indeed, implementing these structures at THz or optical frequencies is challenging. One of the best methods to realize spatiotemporal structures working at higher frequencies of THz and optics, is using graphene. Graphene is a promising material to provide modulation at high frequencies, due to its tunable surface conductivity and compatibility with nanofabrication and integration^33–37^. Additionally, graphene’s response speed is ultrafast: carrier relaxation times are on the order of picoseconds or even femtoseconds^38,39^.

The Optical isolation phenomena of spatiotemporal structures in a silicon slab waveguide with spatially and temporally modulated dielectric, was first analyzed by coupled mode theory (CMT)^1^. This method considers only two fundamental symmetric and asymmetric modes of unperturbed waveguide, which could be effectively converted to each other under the matching conditions of frequency and wave vector provided by spatiotemporal modulated relative permittivity. CMT is a common method for studying space-time modulated structures^4,40–43^, but it has some limitations. CMT uses an approximation of a limited number of participating harmonics to obtain closed-form solutions. However, under certain circumstances, such as strong modulation depth, where the dominant harmonics are not limited to two or three terms, the accuracy of CMT responses decreases significantly^44–46^. Additionally, if there are leaky modes, CMT responses are not reliable. Another approach to analyze spatiotemporal waveguides with TEM or quasi TEM wave responses is the transmission line model^3^. In this technique, the modulated relative permittivity is modeled using distributed time-space periodic shunt capacitances and only three fundamental harmonics are considered in lossless transmission line equations, which limits this method to be used in some applications which require to consider more harmonics^47,48^. To overcome the inherent limitations of CMT, Floquet-Bloch theory was proposed for analyzing structures with spatiotemporal modulated constitutive parameters (permeability and permittivity), which provides complete answers^6,31,49^.

In this work, we extend the use of Floquet-Bloch theory to accurately analyze spatiotemporal modulated graphene-based integrated structures, in order to provide a complete and accurate response and handle both guided and leaky modes precisely, for any modulation parameter. Unlike many previous studies concerned with spatiotemporal modulation of bulk permittivity and/or permeability, here we are engaged with spatiotemporal modulated surface conductivity of the graphene layer. As concrete examples, two typical plasmonic waveguides with both spatially and temporally modulated graphene conductivities are studied to investigate interband transition between different modes and intraband transition in the single mode waveguide. The dependence of mode conversion to the parameters of modulated wave such as direction and modulation depth is thoroughly studied. The accuracy of the proposed analytical method is verified through comparison with the numerical full wave results achieved by using COMSOL Multiphysics software.

We envisage that our study of graphene-based plasmonic waveguides paves the way towards a kind of transceiver front-end. The mere spatial modulation of graphene conductivity enables conversion of a graphene-based plasmonic waveguide to a leaky wave antenna. It follows that the time modulation of graphene conductivity not only changes the frequency of guided wave, but also the frequency of radiated wave. Moreover, the modulation parameters can be changed, to engineer the transmit and receive patterns separately in view of splitting the transmit and receive ports, and to enable the dynamic beam steering. In other words, the spatiotemporal modulation of graphene conductivity allows us to propose a multifunctional device.

The structure of the paper is as follows. First, the analytical model for analysis of spatiotemporal graphene-based structures is introduced and closed-form formulations are developed for two different graphene-based waveguides. Then, the accuracy of the developed formulas is verified through comparison with the results of previously reported works and also numerical results obtained using full wave simulation in COMSOL Multiphysics.

## Analytical modelling and theoretical formulation

Here, we study two different structures, namely parallel plate graphene-based waveguide (PPGW) and plasmonic single mode graphene-based waveguide (SMGW). These plasmonic graphene-based waveguides have been already used as building blocks of different THz devices such as leaky wave antennas, isolators, switches, phase shifters and mode convertors^5,50–53^.

Using Floquet-Bloch theorem, we develop a semi-analytical method for fast and accurate modeling of spatiotemporal graphene-based waveguides. While full wave numerical simulation of the structure is rather complex due to the coupling of many harmonics of the fundamental spatial and temporal frequencies, the simple semi-analytical method determines the electromagnetic fields with almost no computational cost. The semi-analytic method is applicable to various structures with spatiotemporal modulated reactance surfaces which are of prime interest in view of nonreciprocal devices with no external static magnetic fields.

### Parallel plate graphene-based waveguides (PPGW)

**Figure 1:**
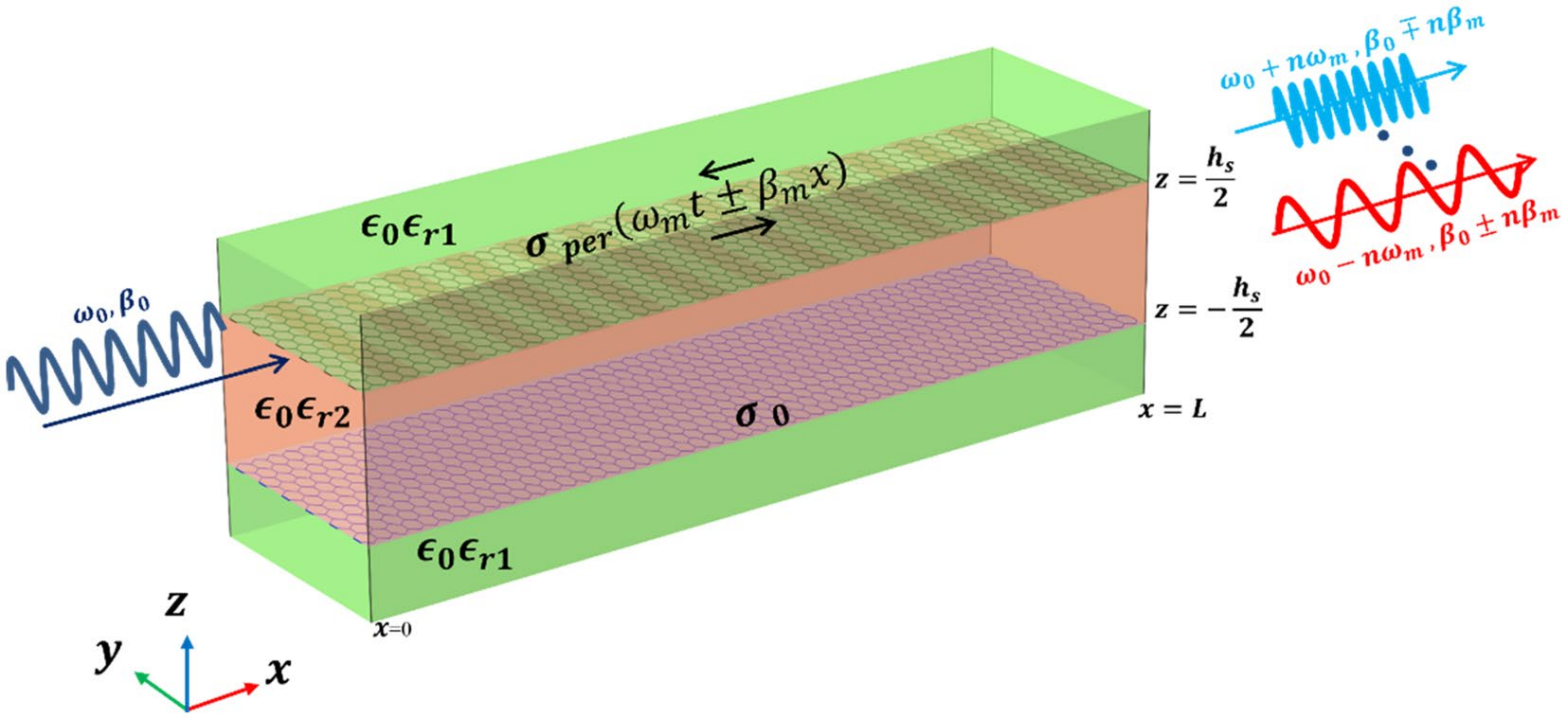
Graphene-based plasmonic waveguide structure. Parallel plate waveguide consists of two graphene layers of infinite width separated by a dielectric of relative permittivity ${\epsilon }_{r2}$ and height ${h}_{s}$.

The investigated graphene-based waveguiding structure is shown in Fig. 1. The structure includes two laterally infinite sheets of graphene, separated by a dielectric of relative permittivity ${\epsilon }_{r2}$ and height ${h}_{s}$. The bottom graphene layer is fabricated on a substrate of relative permittivity ${\epsilon }_{r1}$ and a height much larger than the wavelength. To maintain the symmetry of the structure, the top graphene layer is fabricated on the same substrate. This waveguide supports two fundamental even and odd TM plasmonic modes^54^. Here, we assume that the top graphene layer is periodically modulated both in time and space, while the bottom layer has no modulation. The modulation can be done electronically by applying corresponding voltages to the top graphene layer^55–57^ or optically by using optical pumping^58^. More conveniently, the modulation can be achieved by a guided wave supported by another quasi-TEM waveguide (with a frequency much smaller than the main frequency of PPGW). This modulation directly affects the graphene chemical potential which in turn modifies the graphene surface conductivity^35,59,60^.

The surface conductivity of graphene controlled by its chemical potential in the linear regime and in the absence of magnetic bias can be described by Kubo’s formula including two terms describing interband and intraband carrier transitions^35,59,60^:

1a $$\sigma ={\sigma }_{intra}+{\sigma }_{inter},$$

1b $${\sigma }_{intra}\left(\omega ,{\mu }_{c}\right)=\frac{-j{e}^{2}{k}_{B}T}{\pi {\mathrm{\hslash }}^{2}\left(\omega -j{\tau }^{-1}\right)}\left(\frac{{\mu }_{c}}{{k}_{B}T}+2{\text{ln}}\left({e}^{-\frac{{\mu }_{c}}{{k}_{B}T}}+1\right)\right),$$

1c $${\sigma }_{inter}\left(\omega ,{\mu }_{c}\right)\cong \frac{-j{e}^{2}}{4\pi \hslash }{\text{ln}}\left(\frac{2\left|{\mu }_{c}\right|-\left(\omega -j{\tau }^{-1}\right)\hslash }{2\left|{\mu }_{c}\right|+\left(\omega -j{\tau }^{-1}\right)\hslash }\right),$$

where $e$ is the electron charge, $\hslash$ is the reduced Planck constant,${k}_{B}$ is Boltzmann constant, ${\mu }_{c}$ is the chemical potential, $\tau$ is the relaxation time of graphene, $T$ is the temperature and $\omega$ is the angular frequency.

At THz frequencies where $\omega \hslash <2\left|{\mu }_{c}\right|$, the interband term is negligible^54,61^. Furthermore, as all values of modulated chemical potential in this study are considered far from Dirac point, we assume that $\left|{\mu }_{c}\right|\gg {k}_{B}T$, which allows to employ Drude form of surface conductivity for the intraband term resulting in:

2 $$\sigma \left(\omega ,{\mu }_{c}\right)=\frac{{e}^{2}{\mu }_{c}\tau }{\pi {\hslash }^{2}\left(1+j\omega \tau \right)},$$

As the frequency variation around the working frequency are small, it can be assumed that the frequency dispersion of Drude conductivity can be neglected. Thus, spatiotemporal modulation of chemical potential affects the graphene conductivity, directly. We can express this periodic modulation as a Fourier series:

3 $${\sigma }_{per}=\sum_{q}{\sigma }_{q}{e}^{jq\left({\omega }_{m}t-{\beta }_{m}x\right)},$$

where ${\omega }_{m}$ is the time modulation frequency, ${\beta }_{m}$ is the spatial modulation frequency, and ${\sigma }_{q}$ is the conductivity coefficient corresponding to each harmonic. Particularly, the fundamental harmonic ${(\sigma }_{0})$ is the average surface conductivity of the static graphene.

As Kubo’s formula is valid under local equilibrium condition^62^, graphene conductivity (Eq. (2)) can follow the spatial changes of chemical potential even if the spatial modulation period is as small as few nanometers or even less^13,63,64^. Besides, in high quality graphene with weak disorder and hence low collision frequency, if the modulation frequency of time varying chemical potential is much smaller than the main frequency and plasma resonance frequency, the graphene conductivity can follow the temporal modulation of chemical potential as well^65–67^. Therefore, we can effectively use time modulation frequencies up to THz band.

Having a spatiotemporal modulated graphene layer at the top, makes our structure be periodic both in space and time, thus according to the Floquet–Bloch theorem^68,69^, the electromagnetic response of the system would also be periodic. Therefore, the electromagnetic fields supported by the structure can be represented in terms of Floquet–Bloch modes. Indeed, through the propagation process, the fundamental Floquet mode may convert to other higher order modes and vice versa. Therefore, the amplitude of each harmonic does not remain constant. To account for this effect, and to consider the amplitude changes of each Floquet mode through propagation, we consider the propagation of all harmonics instead of only the fundamental one. The final response is then a combination of all harmonic responses. Therefore, the time domain plasmonic magnetic field, ${\mathcal{H}}_{y}$ of TM waves supported by the waveguide can be expressed in the series form of time harmonics as:

4 $${\mathcal{H}}_{y}\left(x,z,t\right)=\sum_{n=-\infty }^{+\infty }{H}_{y}\left({\omega }_{n},x,z\right){e}^{j{\omega }_{n}t},$$

where ${\omega }_{n}={\omega }_{0}+n{\omega }_{m}$ is the frequency of $n$ th time Floquet harmonic, with ${\omega }_{0}$ being the excitation frequency. ${H}_{y}({\omega }_{n},x,z)$ is the harmonic response of magnetic field at this frequency and can be expressed in three different domains of inside dielectric, above the top graphene layer and below the bottom graphene layer as:

5 $${H}_{y}\left({\omega }_{n},x,z\right)=\sum_{i}{a}_{i}{e}^{-j\left[\beta \left({\omega }_{i}\right)\pm \left(n-i\right){\beta }_{m}\right]x}\left\{\begin{array}{l}{A}_{n}\left({\omega }_{i}\right){e}^{-{\alpha }_{1n,i}z} z\ge \frac{{h}_{s}}{2}\\ \left[{B}_{n}\left({\omega }_{i}\right){\text{cosh}}\left({\alpha }_{2n,i}z\right)+{C}_{n}\left({\omega }_{i}\right){\text{sinh}}\left({\alpha }_{2n,i}z\right)\right] -\frac{{h}_{s}}{2}\le z\le \frac{{h}_{s}}{2} \\ {D}_{n}\left({\omega }_{i}\right){e}^{{\alpha }_{1n,i}z} z\le -\frac{{h}_{s}}{2}\end{array}\right.,$$

where ${A}_{n}\left({\omega }_{i}\right),{B}_{n}\left({\omega }_{i}\right),{C}_{n}\left({\omega }_{i}\right),{D}_{n}({\omega }_{i})$ are the amplitude coefficients of $n$ th harmonic, and $\beta ({\omega }_{i})$ is the propagation constant at the frequency of ${\omega }_{i}$, while ${\alpha }_{1n,i}, {\alpha }_{2n,i}$ are decaying factors of plasmonic waves in the above or below dielectric with the relative permittivity of ${\epsilon }_{r1}$ and middle dielectric with the relative permittivity of ${\epsilon }_{r2}$ and are calculated as:

6a $${\alpha }_{1n,i}=\sqrt{{\left[\beta ({\omega }_{i})\pm (n-i){\beta }_{m}\right]}^{2}-{\epsilon }_{r1}{{K}_{0i}}^{2}},$$

6b $${\alpha }_{2n,i}=\sqrt{{\left[\beta ({\omega }_{i})\pm (n-i){\beta }_{m}\right]}^{2}-{{{\epsilon }_{r2}K}_{0i}}^{2}},$$

where $\left[\beta ({\omega }_{i})\pm (n-i){\beta }_{m}\right]$ is the wave vector of space Floquet harmonic and ${K}_{0i}=\frac{{\omega }_{i}}{c}$. The upper-case sign is used when the propagating and modulation waves are in the same direction (codirectional modulation) while the lower-case sign is used when the propagating and modulation waves are in the opposite direction (contra-directional modulation). The coefficients ${a}_{i}$ in Eq. (5), are calculated using the boundary condition at $x=0$ where only the fundamental harmonic is excited ($\left|H({\omega }_{0},0,\frac{{h}_{s}}{2})\right|=1$, $\left|H({\omega }_{n},0,\frac{{h}_{s}}{2})\right|$=$0$ for $n\ne 0$).

In this formulation, the conversion phenomenon which happens when different Floquet harmonics propagate inside the guiding structure, has been modeled through the series of space harmonics of Eq. (5) and also in the calculation of decay factors shown in Eqs. (6a), (6b). The electric field of the supported modes is then easily calculated using Eq. (5), and Maxwell’s equations.

Now that the formulation of the fields is achieved in the three regions, we need to apply boundary conditions at interfaces of $z=\pm \frac{{h}_{s}}{2}$. To apply appropriate boundary conditions on the frequency domain tangential electric and magnetic field, the phasors ${E}_{x}$ and ${H}_{y}$ at each frequency of ${\omega }_{n}$ are considered. Furthermore, since we have graphene layers at both interfaces, its impact on the discontinuity of tangential component of magnetic fields should be considered in the form of surface current as:

7 $$\widehat{n}\times \left(\overrightarrow{{H}_{t1}}-\overrightarrow{{H}_{t2}}\right)={\sigma }_{s}\overrightarrow{{E}_{t}},$$

where $\widehat{n}$ is the unit vector normal to the graphene sheet and $\overrightarrow{{E}_{t}}$ is the tangential electric phasor and $\overrightarrow{{H}_{t\mathrm{1,2}}}$ are magnetic field phasors of both sides of graphene sheet. It should also be noted that applying boundary conditions on both graphene sheets make the equations for different harmonics to be decoupled, so we drop $i$ indices in the following equations for simplicity. Applying boundary conditions result in:

8a $$\frac{{\alpha }_{1n}}{{\epsilon }_{r1}}{D}_{n}{e}^{-\frac{{{\alpha }_{1n}h}_{s}}{2}}=\frac{{\alpha }_{2n}}{{\epsilon }_{r2}}\left[-{B}_{n}{\text{sinh}}\left(\frac{{{\alpha }_{2n}h}_{s}}{2}\right)+{C}_{n}{\text{cosh}}\left(\frac{{{\alpha }_{2n}h}_{s}}{2}\right)\right],$$

8b $$\left(\frac{{\alpha }_{1n}{\sigma }_{0}}{j{\omega }_{n}{\epsilon }_{0}{\epsilon }_{r1}}+1\right){D}_{n}{e}^{\frac{{-{\alpha }_{1n}h}_{s}}{2}}={B}_{n}{\text{cosh}}\left(\frac{{{\alpha }_{2n}h}_{s}}{2}\right)+{C}_{n}{\text{sinh}}\left(\frac{{{\alpha }_{2n}h}_{s}}{2}\right),$$

8c $$\frac{{\alpha }_{1n}}{{\epsilon }_{r1}}{A}_{n}{e}^{\frac{{-{\alpha }_{1n}h}_{s}}{2}}=\frac{-{\alpha }_{2n}}{{\epsilon }_{r2}}\left[{B}_{n}{\text{sinh}}\left(\frac{{{\alpha }_{2n}h}_{s}}{2}\right)+{C}_{n}{\text{cosh}}\left(\frac{{{\alpha }_{2n}h}_{s}}{2}\right)\right].$$

8d $${A}_{n}{e}^{\frac{{-{\alpha }_{1n}h}_{s}}{2}}-{B}_{n}{\text{cosh}}\left(\frac{{{\alpha }_{2n}h}_{s}}{2}\right)-{C}_{n}{\text{sinh}}\left(\frac{{{\alpha }_{2n}h}_{s}}{2}\right)=-\sum_{q}{\sigma }_{q}\frac{{\alpha }_{1n-q}}{j{\omega }_{n-q}{\epsilon }_{0}{\epsilon }_{r1}}{A}_{n-q}{e}^{\frac{{-{\alpha }_{1n-q}h}_{s}}{2}}.$$

Combing these equations results in a recursive relation for each harmonic as:

9 $$\left[1+\frac{{\alpha }_{1n}{\epsilon }_{r2}}{{\alpha }_{2n}{\epsilon }_{r1}}\frac{{c}_{n}{\text{cosh}}\left({\alpha }_{2n}{h}_{s}\right)+{\text{sinh}}\left({\alpha }_{2n}{h}_{s}\right)}{{\text{cosh}}\left({\alpha }_{2n}{h}_{s}\right)+{c}_{n}{\text{sinh}}\left({\alpha }_{2n}{h}_{s}\right)}+\frac{{{\sigma }_{0}\alpha }_{1n}}{j{\omega }_{n}{\epsilon }_{0}{\epsilon }_{r1}}\right]{e}^{\frac{{-{\alpha }_{1n}h}_{s}}{2}}{A}_{n}+\sum_{q}\frac{{{\sigma }_{q}\alpha }_{1n-q}}{j{\omega }_{n-q}{\epsilon }_{0}{\epsilon }_{r1}}{e}^{\frac{{-{\alpha }_{1n-q}h}_{s}}{2}}{A}_{n-q}=0,$$

with $${c}_{n}=\left(1+\frac{{\sigma }_{0}{\alpha }_{1n}}{j{\omega }_{n}{\epsilon }_{0}{\epsilon }_{r1}}\right)\frac{{\alpha }_{2n}{\epsilon }_{r1}}{{\alpha }_{1n}{\epsilon }_{r2}}$$.

For the convergence condition to be satisfied for these infinite set of linear homogenous equations of infinite unknowns $\left|{A}_{n}\right|$ s should be uniformly decreasing. Thus, the number of equations can be truncated to $2N+1$ (from $-N$ to $N$) and remaining equations can be represented in the matrix form of $\left[K\right]\left[A\right]=0$, where $\left[A\right]$ is the vector matrix of ${A}_{n}$ s, and $\left[K\right]$ is the square matrix of coefficient with elements of:

10 $${k}_{mk}=\left\{\begin{array}{l}\left[1+\frac{{\alpha }_{1m}{\epsilon }_{r2}}{{\alpha }_{2m}{\epsilon }_{r1}}\frac{{c}_{m}{\text{cosh}}\left({\alpha }_{2m}{h}_{s}\right)+{\text{sinh}}\left({\alpha }_{2m}{h}_{s}\right)}{{\text{cosh}}\left({\alpha }_{2m}{h}_{s}\right)+{c}_{m}{\text{sinh}}\left({\alpha }_{2m}{h}_{s}\right)}+\frac{{{\sigma }_{0}\alpha }_{1m}}{j{\omega }_{m}{\epsilon }_{0}{\epsilon }_{r1}}\right]{e}^{\frac{{-{\alpha }_{1m}h}_{s}}{2}} k=m\\ \frac{{{\sigma }_{m-k}\alpha }_{1k}}{j{\omega }_{k}{\epsilon }_{0}{\epsilon }_{r1}}{e}^{\frac{{-{\alpha }_{1k}h}_{s}}{2}} k\ne m\end{array}\right..$$

Finally, the dispersion equation can be achieved by applying the ${\text{det}}\left(K\right)=0$ which is the condition to have nontrivial solution of the above equations. After finding $\beta ({\omega }_{i})$, using the dispersion equation, all ${A}_{n}$ s can be obtained in terms of the amplitude of the fundamental harmonic, ${A}_{0}$.

For the case of sinusoidally modulated graphene, which is one of the most practical cases used in different applications^1,2,5,7,8,31^, the conductivity of the graphene can be written as:

11 $$\sigma ={\sigma }_{0}\left(1+M{\text{cos}}\left({\omega }_{m}t\pm {\beta }_{m}x\right)\right)={\sigma }_{0}+\frac{{\sigma }_{0}M}{2}{e}^{j\left({\omega }_{m}t\pm {\beta }_{m}x\right)}+\frac{{\sigma }_{0}M}{2}{e}^{-j\left({\omega }_{m}t\pm {\beta }_{m}x\right)},$$

where $M$ is the modulation depth and ${\sigma }_{0}$ is the average conductivity. In this case, the recursive formulation of Eq. (9) will result in the following equations:

12a $${a}_{n}{A}_{n}+{b}_{n-1}{A}_{n-1}+{b}_{n+1}{A}_{n+1}=0,$$

12b $${a}_{n}=\left[1+\frac{{\alpha }_{1n}{\epsilon }_{r2}}{{\alpha }_{2n}{\epsilon }_{r1}}\frac{{c}_{n}{\text{cosh}}\left({\alpha }_{2n}{h}_{s}\right)+{\text{sinh}}\left({\alpha }_{2n}{h}_{s}\right)}{{\text{cosh}}\left({\alpha }_{2n}{h}_{s}\right)+{c}_{n}{\text{sinh}}\left({\alpha }_{2n}{h}_{s}\right)}+\frac{{{\sigma }_{0}\alpha }_{1n}}{j{\omega }_{n}{\epsilon }_{0}{\epsilon }_{r1}}\right]{e}^{\frac{{-{\alpha }_{1n}h}_{s}}{2}},$$

12c $${b}_{n}=\frac{{\sigma }_{0}M}{2}\frac{{\alpha }_{1n}}{j{\omega }_{n}{\epsilon }_{0}{\epsilon }_{r1}}{e}^{\frac{{-{\alpha }_{1n}h}_{s}}{2}}.$$

The unknown coefficients ${A}_{n}$ can be found in term of ${A}_{0}$, by continued fractions as:

13 $${A}_{n}=\left\{\begin{array}{c}{A}_{n+1}\frac{{b}_{n+1}}{-{a}_{n}+\frac{{b}_{n}{b}_{n-1}}{{a}_{n-1}+\frac{{b}_{n-1}{b}_{n-2}}{-{a}_{n-2}+\frac{{b}_{n-2}{b}_{n-3}}{{a}_{n-3}+ \dots }}}} n<0\\ {A}_{n-1}\frac{{b}_{n-1}}{-{a}_{n}+\frac{{b}_{n}{b}_{n+1}}{{a}_{n+1}+\frac{{b}_{n+1}{b}_{n+2}}{-{a}_{n+2}+\frac{{b}_{n+2}{b}_{n+3}}{{a}_{n+3}+ \dots }}}} n>0\end{array}\right..$$

By substituting ${A}_{1}$ and ${A}_{-1}$ from Eq. (13) into Eq. (12a) for $n=0$, the dispersion equation is achieved as:

14 $${a}_{0}+\frac{{b}_{-1}{b}_{0}}{-{a}_{-1}+\frac{{b}_{-1}{b}_{-2}}{{a}_{-2}+\frac{{b}_{-2}{b}_{-3}}{-{a}_{-3}+\frac{{b}_{-3}{b}_{-4}}{{a}_{-4}+ \dots }}}}+\frac{{b}_{1}{b}_{0}}{-{a}_{1}+\frac{{b}_{1}{b}_{2}}{{a}_{2}+\frac{{b}_{2}{b}_{3}}{-{a}_{3}+\frac{{b}_{3}{b}_{4}}{{a}_{4}+ \dots }}}}=0.$$

### Single mode graphene-based waveguide (SMGW)

**Figure 2:**
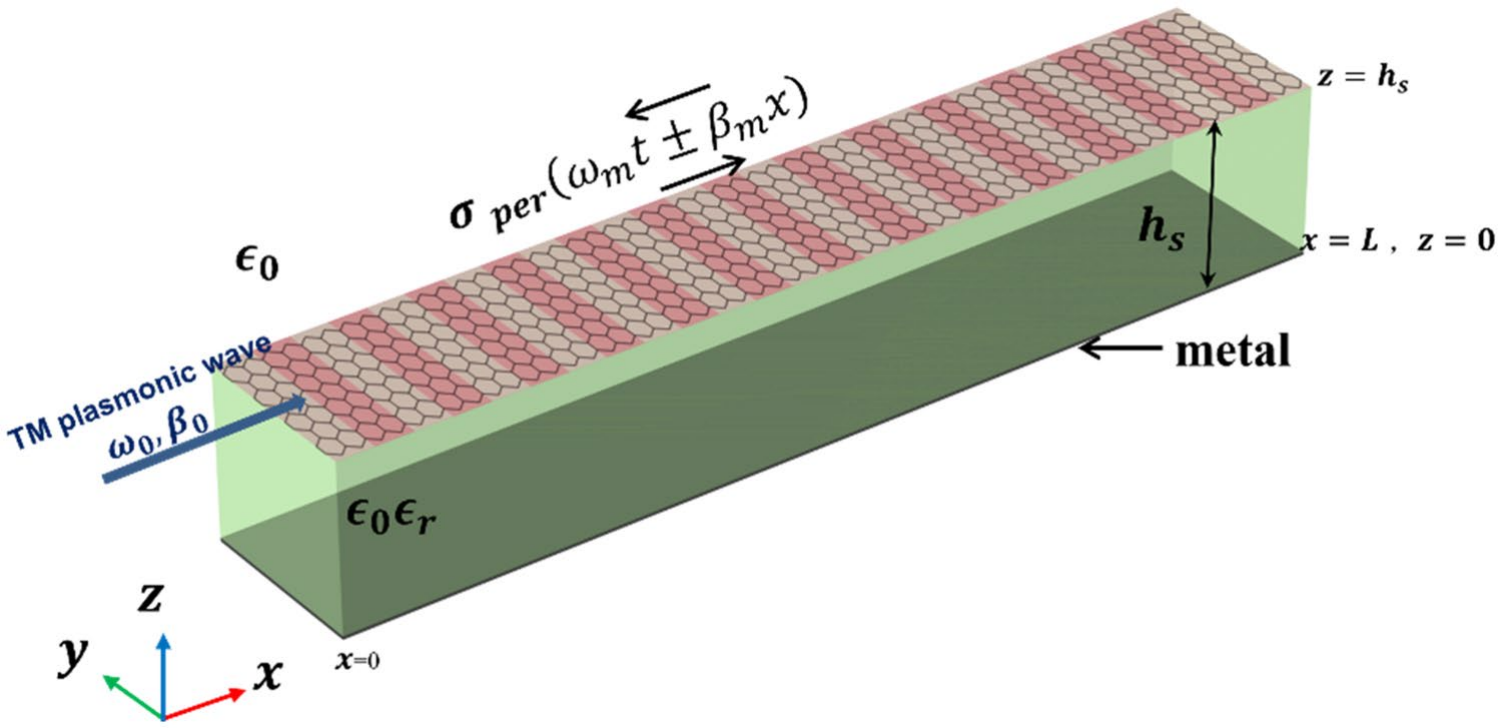
Single mode graphene-based plasmonic waveguide made of a graphene layer on top of metal backed substrate of relative permittivity of ${\epsilon }_{r}$ and height ${h}_{s}$.

The second structure which is a single mode graphene plasmonic waveguide (SMGW) is shown in Fig. 2. This graphene-based waveguide is composed of a laterally infinite graphene layer above a grounded dielectric substrate.

Assuming that the conductivity of the graphene layer is modulated as Eq. (3), the analysis would be very similar to the previous section with Floquet-Bloch series of electric and magnetic fields, except for the boundary condition dictated by the metallic layer at the bottom of the structure. So, it yields the recursive relation as:

15 $$\left[1+\frac{{\alpha }_{1n}{\epsilon }_{r}}{{\alpha }_{2n}}\mathit{cot}h\left({\alpha }_{2n}{h}_{s}\right)\right]{A}_{n}+\sum_{q}\frac{{{\sigma }_{q}\alpha }_{1n-q}}{j{\omega }_{n-q}{\epsilon }_{0}}{A}_{n-q}=0.$$

consequently, the elements of coefficient matrix $\left[K\right]$ will be

16 $${k}_{mk}=\left\{\begin{array}{l}1+\frac{{\alpha }_{1m}{\epsilon }_{r}}{{\alpha }_{2m}}\mathit{cot}h\left({\alpha }_{2m}{h}_{s}\right)+\frac{{{\sigma }_{0}\alpha }_{1m}}{j{\omega }_{m}{\epsilon }_{0}} k=m\\ \frac{{{\sigma }_{m-k}\alpha }_{1k}}{j{\omega }_{k}{\epsilon }_{0}} k\ne m\end{array}\right.,$$

In the case of sinusoidally modulation, the coefficients of the three-term recursive of Eq. (12a) will be

17a $${a}_{n}=1+\frac{{\alpha }_{1n}{\epsilon }_{r}}{{\alpha }_{2n}}\mathit{cot}h\left({\alpha }_{2n}{h}_{s}\right)+\frac{{{\sigma }_{0}\alpha }_{1n}}{j{\omega }_{n}{\epsilon }_{0}},$$

17b $${b}_{n}=\frac{{\sigma }_{0}M}{2}\frac{{\alpha }_{1n}}{j{\omega }_{n}{\epsilon }_{0}}.$$

## Results and discussion

To investigate the accuracy of our Floquet-Bloch theory-based approach, here we compare our analytical results with full wave numerical simulation results obtained via COMSOL Multiphysics. PPGW and SMGW structures shown in Figs. 1 and 2 are studied. The dimensions and parameters characterizing the systems are listed in Table 1. The modulation of the graphene conductivity is assumed to be sinusoidal as in Eq. (11). Concerning PPGW, we reproduced Fig. 2a and b of Ref. 5 to examine our analytical approach.

**Table 1:** The parameters used in the structures of Figs. 1, 2.

	${h}_{s}[\mu m]$	${\epsilon }_{r1} , {\epsilon }_{r2}$	${f}_{0}[THz]$	${f}_{m}[THz]$	${\lambda }_{m}[\mu m]$	$M$	${\mu }_{c}[eV]$	$\tau [ps]$
PPGW-case1	$1$	$1 , 1$	$10$	$1$	$11.07$	$0.1$	$0.5$	$1$
PPGW-case2	0.5	2.5, 2	10	0.7	6	0.1	0.5	1
SMGW	$5$	$3.8$	$3$	$0.3$	$200$	$0.1$	$0.6$	$1$

A remark on the modulation depth ($M$) is in order. As a first approximation, graphene chemical potential can be written as^70^ ${\mu }_{c}=\hslash {v}_{F}\sqrt{\pi \eta \left|{V}_{g}+{V}_{offset}\right|}$, where $\hslash$ is the reduced Planck constant, ${v}_{F}\sim {10}^{6}\frac{m}{s}$ is the Fermi velocity, ${V}_{offset}$ is the offset voltage caused by natural doping, ${V}_{g}$ is bias voltage and $\eta$ can be estimated using a parallel plate capacitor model. For a typical chemical potential ${\mu }_{c0}=0.5 eV$ and a typical $100nm$ distance between the graphene layer and the ground plane, one finds ${V}_{offset }=87.5 V$. Variation of bias voltages about $\pm 18.4 V$ results in chemical potentials between 0.45 and $0.55 eV$. It follows that a modulation depth $M=0.1$ is not out of reach.

**Figure 3:**
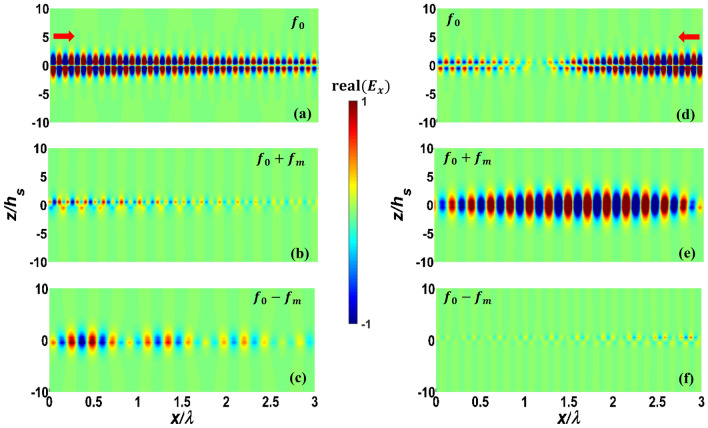
Real part of normalized electric field ${E}_{x}$ of modulated PPGW-case1. (**a**–**c**) codirectional case and (**d**–**f**) contra-directional case of fundamental harmonic at ${f}_{0}$ and first harmonics at ${f}_{0}\pm {f}_{m}$ (the structure is excited at the frequency of ${f}_{0}$).

First, we consider PPGW-case1 with dimensions and parameters shown in Table 1. In previous works^5^, it has been shown that this structure can be used as an isolator: when the propagating and modulation waves are codirectional, most of the power will remain in the excited frequency of ${f}_{0}$ , while in the case of contra-direction, power of fundamental harmonic with the frequency of ${f}_{0}$ will be mostly coupled to the wave with frequency of ${f}_{0}+{f}_{m}$, and a non-remarkable power at the frequency of ${f}_{0}$ will reach to the end of the waveguide^5^. Here, we use our analytical model to investigate and study this phenomenon. The results of this analysis are shown in Fig. 3 and also Table 2.

**Table 2:** ${A}_{n}$ Coefficients of nth harmonics normalized to ${A}_{0}$ and ${n}_{eff}$ for structures with dimensions shown in Table 1.

$n$	Contra-directional modulated PPGW ${n}_{eff}=7.48-0.09i$	Codirectional modulated PPGW ${n}_{eff}=7.36-0.081i$
$-5$	$0.000001$	$0.00000008$
$-4$	$0.00002$	$0.000001$
$-3$	$0.0004$	$0.0002$
$-2$	$0.0066$	$0.009$
− $1$	$0.0915$	$0.1072$
1	$1.2796$	$0.1544$
$2$	$0.0568$	$0.01937$
$3$	$0.0006$	$0.0022$
$4$	$0.00001$	$0.0002$
$5$	$0.000002$	$0.00002$

Figure 3 illustrates the real part of longitudinal electric field ${E}_{x}$ , calculated using magnetic field of Eq. (5) and Maxwell’s equations, of the waves propagating inside the modulated parallel plate waveguide for both codirectional (Fig. 3a–c) and contra-directional (Fig. 3d–f) cases. The non-reciprocal response of the spatiotemporal modulated PPGW (its directional response) is apparent from the results of Fig. 3. Excited Odd mode at ${f}_{0}$ is substantially coupled to the Even mode at ${f}_{0}+{f}_{m}$ in contra-directional modulation (see Fig. 3e), while no significant mode conversion occurs in the codirectional case (see Fig. 3b,c). As wave propagates through spatiotemporal modulated structure and experiences frequency and propagation constant conversions, the power exchange between harmonics occurs periodically. Coherence length is a typical parameter to estimate the length of the device in which the most of the power is delivered to other harmonics from the fundamental one. Power exchange and coherence length can be calculated with the proposed analytical model. As shown in Fig. 3d, in the contra-directional modulation, the amplitude of the fundamental harmonic is at its lowest level at the coherence length of ${L}_{c}=1.86\lambda =56\mu m$ and most of the power is transfered to the 1th harmonic. After this length, the power will return to the fundamental mode gradually and this process is repeated periodically. This determines an optimum length when designing isolators using these spatiotemporal structures. The results shown in Fig. 3 confirm the findings reported in Ref.^5^.

To have more clarification on the results shown in Fig. 3, more details are illustrated in Table 2. In this table, the middle results achieved in the analytical modelling are presented. One of the results presented in Table 2, is the effective refractive index of the spatiotemporal modulated waveguide, ${n}_{eff}$, which is calculated using the dispersion relation presented in Eq. (14). This value has been calculated and illustrated both for contra-directional and codirectional cases. This table also illustrates the normalized amplitude coefficients, ${A}_{n}$ for ten different harmonics; $n=-5:5$. The results of Table 2 exhibit a non-reciprocal response for the modulated PPWG waveguide as also reported in Ref.^5^. As shown in this table, in contra-directional modulation, the amplitude of the first harmonic, ${A}_{1}$ is much bigger than any other harmonics, while in the codirectional case, none of the harmonics has a significant amplitude, meaning that in this case no modulation happens and the power remains at the excitation frequency of ${f}_{0}$ as also illustrated in Fig. 3.

**Figure 4:**
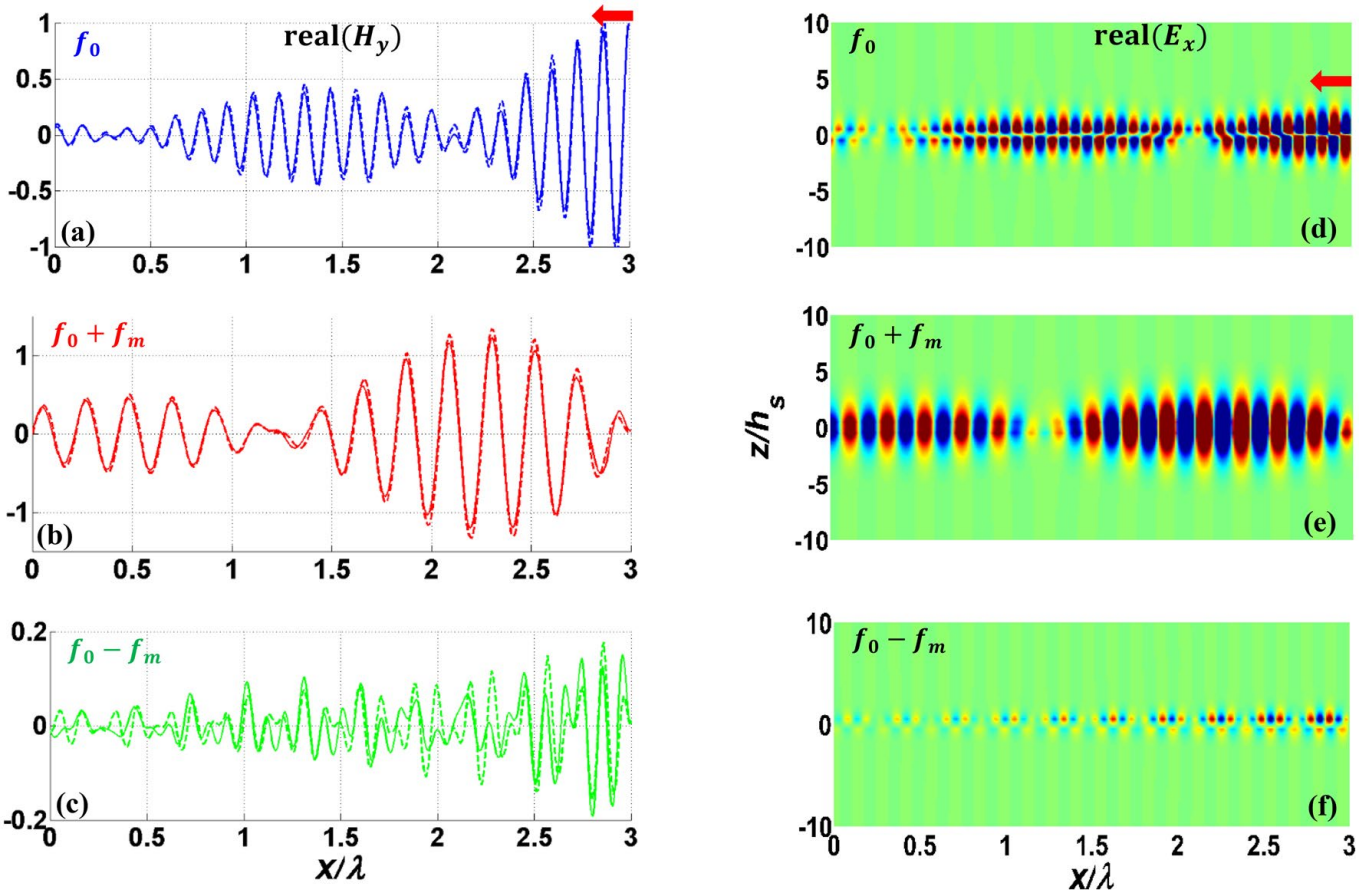
Real part of 1D normalized ${H}_{y}$(**a**-**c**) and 2D normalized ${E}_{x}$ (**d**-**f**) for fundamental harmonic(**a**,**d**) and two first harmonics (**b**,**c**,**e**,**f**) of contra-directional modulated PPGW with stronger modulation depth of $M=0.2$. Dashed lines show the results obtained using our analytical model and solid lines illustrate COMSOL results.

By stronger modulation (larger modulation depth, $M$), it is expected that more power be transferred at a shorter coherence length. To verify this prediction and also to test our analytical model for more examples, we study another parallel plate waveguide, this time with higher modulation depth. In this study, $M$ has been increased to 0.2, and other parameters are kept the same as shown in Table 1 (PPGW-case1). The results of this study are shown in Fig. 4. This figure illustrates both 2D and 1D results. As shown in this figure, the coherence length would be equal to ${L}_{c}=0.9\lambda$ which is much shorter than the case with the lower modulation depth we studied earlier. This confirms the prediction that states by increasing the modulation depth, one can design isolators with shorter lengths. The results of Fig. 4, also indicates the wave amplification due to the power transfer from both fundamental mode of signal wave and modulating wave to the desired mode. Travelling wave parametric amplification is one of the first and well known properties of spatiotemporal waveguides already reported in previous works^71,72^. With the purpose of amplification, it is better to choose the waveguide length equal to the coherence length.

**Figure 5:**
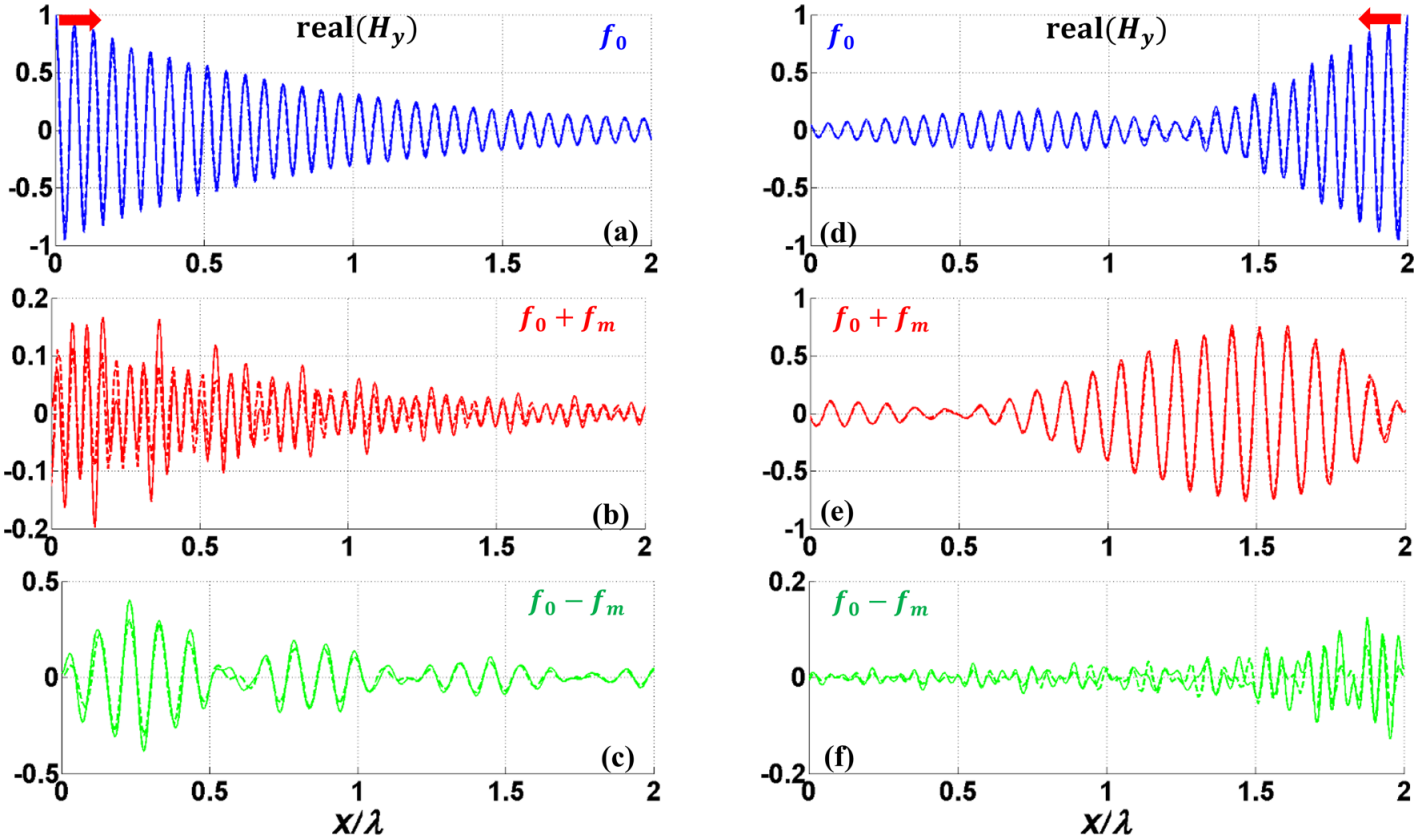
Real part of normalized magnetic field, ${H}_{y}$ of PPGW-case2 at modulated graphene sheet boundary. (**a**–**c**) are for codirectional and (**d**–**f**) are for contra-directional modulation. (**a**,**d**) fundamental harmonic, (**b**,**e**) $n=1$, (**c**,**f**) $n=-1$. Dashed lines show the results obtained using our analytical model and solid lines illustrate COMSOL results.

To verify the accuracy of our analytical model further, different dielectric materials are considered below and above graphene layers with dimensions and parameters shown in Table 1 (PPGW-case2). Here we compare our analytical results with the numerical full wave results achieved by performing simulation in COMSOL Multiphysics. This comparison is shown in Fig. 5, in which the magnetic field at the modulated graphene sheet (the upper layer) is illustrated. In the numerical simulation, coupled physics are defined for each frequency harmonic and modulated graphene is modeled using space variable surface current density in each physics. More detailed information of simulation setup is presented in section “Method”. As shown in Fig. 5, a great agreement is observed between our analytical model and the numerical results achieved from COMSOL, confirming the accuracy of the developed model. Furthermore, our analytical model is about four times faster than the numerical simulation, using the same computational resources.

**Figure 6:**
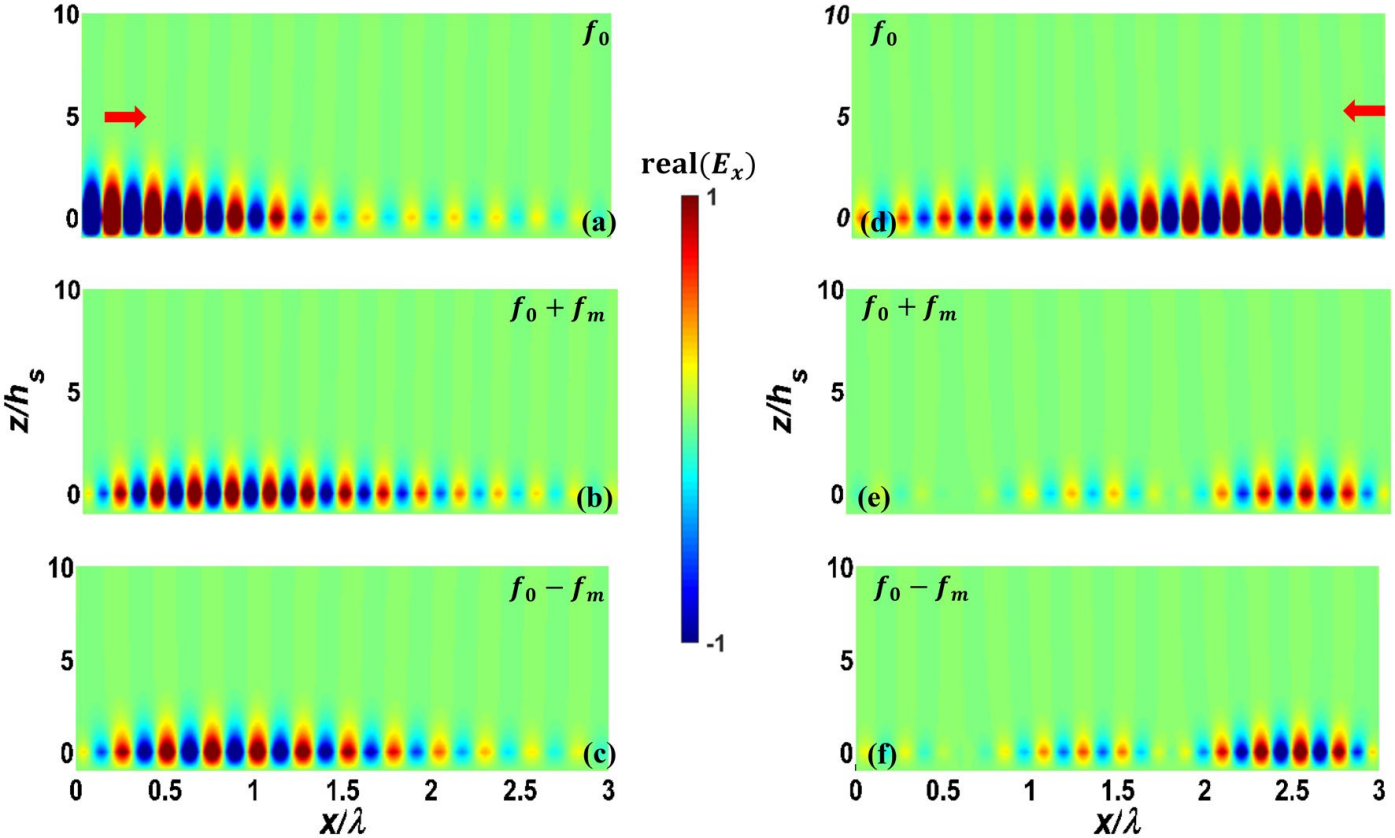
Real part of normalized electric field ${E}_{x}$ of modulated SMGW. (**a**–**c**) codirectional case and (**d**–**f**) contra-directional case of fundamental harmonic at ${f}_{0}$ and first harmonics at ${f}_{0}\pm {f}_{m}$. The excitation frequency is ${f}_{0}$.

**Figure 7:**
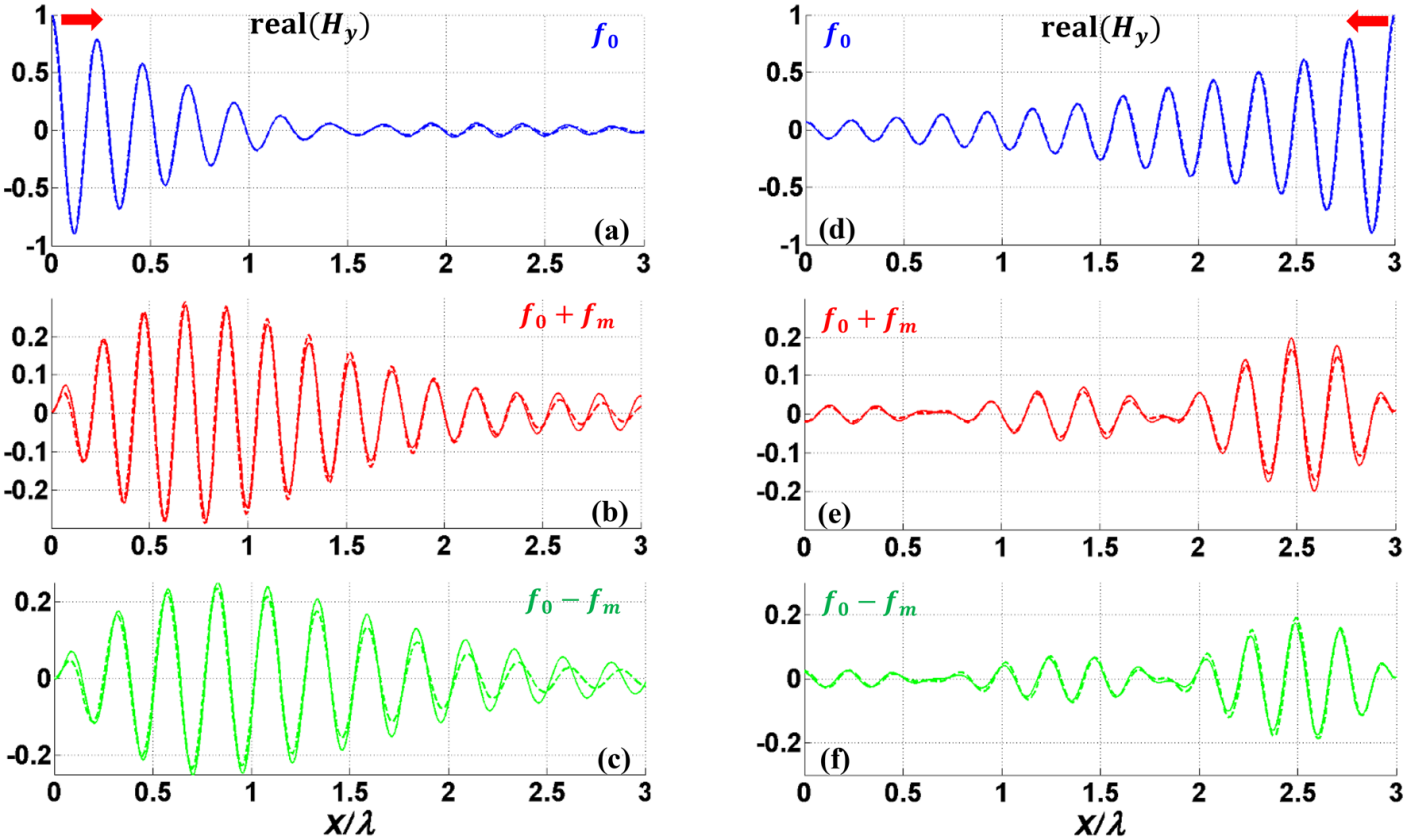
Real part of normalized magnetic field ${H}_{y}$ of SMGW at modulated graphene sheet boundary. (**a**–**c**) are for codirectional and (**d**–**f**) are for contra-directional modulation. (**a**,**d**) fundamental harmonic, (**b**,**e**) $n=1$, (c,f) $n=-1$. Dashed lines are obtained from our analytical model and solid lines are from COMSOL results.

Now we study SMGW structure shown in Fig. 2 with the geometrical and physical parameters listed in Table 1. The results of this study are shown in Figs. 6, 7, and also Table 3. In the single mode structure, spatiotemporal modulation can cause intraband transition which changes the frequency and propagation constant along the dispersion diagram of one mode. Figure 6 illustrates 2D results of the first three harmonics. As shown in this figure, coupling is still dependent to modulation direction and in the codirectional case, most of the power of the fundamental harmonic exchanges to other harmonics at the coherence length of ${L}_{c}=1.6\lambda =160\mu m$. However, this conversion to counterpart harmonics is almost equal and multiple harmonics are excited (as also illustrated in Table 3). It is owing to the fact that with this small modulation frequency, we remain close to the main frequency on the dispersion diagram and the wave experiences little dispersion, thus it is inevitable to excite other harmonics. In order to have more accurate results in Figs. 6 and 7, more harmonics have been contributed in the series of Eq. (5) for SMGW than PPGW. Figure 7 illustrates the magnetic field of three first harmonics on the graphene layer and compares it with numerical results achieved in COMSOL. Here, also we observe a very good agreement between the analytical and numerical results verifying the accuracy of the developed analytical model.

**Table 3:** ${A}_{n}$ Coefficients of nth harmonics normalized to ${A}_{0}$ and ${n}_{eff}$ for parameters of Table 1.

$n$	Contra-directional modulated SMGW ${n}_{eff}=4.33-0.138i$	Codirectional modulated SMGW ${n}_{eff}=4.36-0.144i$
$-5$	$0.000003$	$0.0003$
$-4$	$0.00006$	$0.0059$
$-3$	$0.001$	$0.0652$
$-2$	$0.0145$	$0.3954$
− $1$	$0.1565$	$1.1793$
1	$0.1465$	$1.4136$
$2$	$0.0067$	$0.8823$
$3$	$0.00007$	$0.3963$
$4$	$0.0000009$	$0.1477$
$5$	$0.00000004$	$0.0486$

By increasing the modulation depth, the coherence length can be shortened but simultaneously more power will be transferred to higher order harmonics. In contra-directional case, the results show no significant power coupling and the reduction of the amplitude of fundamental mode is mostly due to plasmonic wave attenuation.

In order to have an accurate comparison between our analytical results and the simulation results, modulation parameters are chosen so that no leaky mode is excited in lower order harmonics. However, our analysis is comprehensive and can be unconditionally used for leaky waves as well.

The PPGW structure (Fig. 1) could be fabricated in the following way^73,74^: The bottom substrate with relative permittivity ${\epsilon }_{r1}$ is deposited on a silicon wafer. Surface planarization or spin-coating of an extra thin layer improves the smoothness of the substrate surface. The chemical vapor deposition (CVD)-grown graphene on copper is then transferred to the smooth substrate. The middle layer with relative permittivity ${\epsilon }_{r2}$ can be deposited via atomic layer deposition (ALD) or plasma-enhanced chemical vapor deposition (PECVD) methods. The spin-coating process can be utilized for polymeric materials. The second graphene layer is transferred to the middle layer. The top layer with relative permittivity ${\epsilon }_{r1}$ is deposited. A similar procedure can be employed to fabricate SMGW (Fig. 2): Via ALD or PECVD methods, a dielectric layer with relative permittivity ${\epsilon }_{r}$ is deposited on a back metalized silicon wafer. The graphene is then transferred to the substrate.

## Conclusion

Graphene-based spatiotemporal modulated structures are promising for different kinds of applications such as isolators, parametric amplifiers and leaky wave antennas. We have presented a Floquet-Bloch theory-based semi-analytical method for analysis of structures involving spatiotemporal modulated surface conductivities. While full wave numerical simulation of modulated structures is rather complex due to the coupling of many harmonics of the fundamental spatial and temporal frequencies, the semi-analytical method determines the electromagnetic fields with almost no computational cost.

We have studied the behavior of two different structures, namely parallel plate graphene-based waveguide (PPGW) and plasmonic single mode graphene-based waveguide (SMGW). The results are verified through comparison with full wave numerical simulations. We show extraordinary spatiotemporal effects such as nonreciprocity, mode conversion and amplification for these structures. We envisage that our study paves the way towards a kind of transceiver front-end. Indeed, the spatial modulation of graphene conductivity enables conversion of a graphene-based plasmonic waveguide to a leaky wave antenna. The time modulation of graphene conductivity allows to engineer the transmit and receive patterns separately and to enable the dynamic beam steering. In other words, the spatiotemporal modulation of graphene conductivity allows us to propose a multifunctional device.

## Methods

The numerical results are obtained using full wave simulation by COMSOL Multiphysics. Particularly, three Frequency Domain Physics interfaces for three frequencies ${f}_{0},{f}_{0}+{f}_{m},{f}_{0}-{f}_{m}$ are coupled together. In the first Frequency Domain Physics interface, the left (right) boundary, associated with codirectional (contra-directional) case mentioned before, is assigned to be user defined port. This allows excitation of the structure with the appropriate plasmonic wave. Other external boundaries are defined as scattering boundary conditions.

Material properties at three frequencies ${f}_{0},{f}_{0}+{f}_{m},{f}_{0}-{f}_{m}$ are properly taken into account. The surface current density of the graphene layer depends on the space–time modulated conductivity of the graphene and the tangential component of the electric field. This naturally couples Frequency Domain Physics interfaces (for example, an electric field term of frequency ${f}_{0}-{f}_{m}$ and a conductivity term of frequency ${f}_{m}$ result in a surface current density term of frequency ${f}_{0}$).

## Data Availability

The dataset used and/or analyzed during the current study are available from the corresponding author on reasonable request.

## References

[1] Yu Z, Fan S (2009). Complete optical isolation created by indirect interband photonic transitions. Nat. Photonics.

[2] Lira H, Yu Z, Fan S, Lipson M (2012). Electrically driven nonreciprocity induced by interband photonic transition on a silicon chip. Phys. Rev. Lett..

[3] Qin S, Xu Q, Wang YE (2014). Nonreciprocal components with distributedly modulated capacitors. IEEE Trans. Microw. Theory Tech..

[4] Estep NA, Sounas DL, Soric J, Alu A (2014). Magnetic-free non-reciprocity and isolation based on parametrically modulated coupled-resonator loops. Nat. Phys..

[5] Correas-Serrano D (2015). Nonreciprocal graphene devices and antennas based on spatiotemporal modulation. IEEE Antennas Wirel. Propag. Lett..

[6] Taravati S, Chamanara N, Caloz C (2017). Nonreciprocal electromagnetic scattering from a periodically space-time modulated slab and application to a quasisonic isolator. Phys. Rev. B.

[7] Guo X, Ding Y, Duan Y, Ni X (2019). Nonreciprocal metasurface with space–time phase modulation. Light Sci. Appl..

[8] Hadad Y, Soric JC, Alu A (2016). Breaking temporal symmetries for emission and absorption. Proc. Natl. Acad. Sci..

[9] Taravati S, Khan BA, Gupta S, Achouri K, Caloz C (2017). Nonreciprocal nongyrotropic magnetless metasurface. IEEE Trans. Antennas Propag..

[10] Chamanara N, Taravati S, Deck-Léger Z-L, Caloz C (2017). Optical isolation based on space-time engineered asymmetric photonic band gaps. Phys. Rev. B.

[11] Taravati S (2018). Aperiodic space-time modulation for pure frequency mixing. Phys. Rev.B.

[12] Shi B (2016). Tunable band-stop filters for graphene plasmons based on periodically modulated graphene. Sci. Rep..

[13] Tu NH (2020). Active spatial control of terahertz plasmons in graphene. Commun. Mater..

[14] Tu NH, Takamura M, Ogawa Y, Suzuki S, Kumada N (2018). Plasmon confinement by carrier density modulation in graphene. Jpn. J. Appl. Phys..

[15] Shameli MA, Fallah A, Yousefi L (2021). Developing an optimized metasurface for light trapping in thin-film solar cells using a deep neural network and a genetic algorithm. J. Opt. Soc. Am. B.

[16] Salami P, Yousefi L (2020). Wide-band polarisation-independent metasurface-based carpet cloak. IET Microw. Antennas Propag..

[17] Akbari-Chelaresi H, Salami P, Yousefi L (2022). Far-field sub-wavelength imaging using high-order dielectric continuous metasurfaces. Opt. Express.

[18] Menendez GA, Maes B (2017). Time reflection and refraction of graphene plasmons at a temporal discontinuity. Opt. Lett..

[19] Wilson J, Santosa F, Min M, Low T (2018). Temporal control of graphene plasmons. Phys. Rev. B.

[20] Shirokova A, Maslov A, Bakunov M (2019). Scattering of surface plasmons on graphene by abrupt free-carrier generation. Phys. Rev. B.

[21] Abed O, Yousefi L (2020). Tunable metasurfaces using phase change materials and transparent graphene heaters. Opt. Express.

[22] Dötsch H (2005). Applications of magneto-optical waveguides in integrated optics. J. Opt. Soc. Am. B.

[23] Gurevich AG, Melkov GA (2020). Magnetization Oscillations and Waves.

[24] Soljačić M, Luo C, Joannopoulos JD, Fan S (2003). Nonlinear photonic crystal microdevices for optical integration. Opt. Lett..

[25] Fan L (2012). An all-silicon passive optical diode. Science.

[26] Mahmoud AM, Davoyan AR, Engheta N (2015). All-passive nonreciprocal metastructure. Nat. Commun..

[27] Shi Y, Yu Z, Fan S (2015). Limitations of nonlinear optical isolators due to dynamic reciprocity. Nat. Photonics.

[28] Sounas DL, Alù A (2018). Fundamental bounds on the operation of Fano nonlinear isolators. Phys. Rev. B.

[29] Estep NA, Sounas DL, Alù A (2016). Magnetless microwave circulators based on spatiotemporally modulated rings of coupled resonators. IEEE Trans. Microwave Theory Techn..

[30] Kord A, Sounas DL, Alù A (2017). Magnet-less circulators based on spatiotemporal modulation of bandstop filters in a delta topology. IEEE Trans. Microwave Theory Techn..

[31] Taravati S, Caloz C (2016). Mixer-duplexer-antenna leaky-wave system based on periodic space-time modulation. IEEE Trans. Antennas Propag..

[32] Horie Y, Arbabi A, Arbabi E, Kamali SM, Faraon A (2017). High-speed, phase-dominant spatial light modulation with silicon-based active resonant antennas. Acs Photonics.

[33] Geim AK, Novoselov KS (2007). The rise of graphene. Nat. Mater..

[34] Bonaccorso F, Sun Z, Hasan T, Ferrari A (2010). Graphene photonics and optoelectronics. Nat. Photonics.

[35] Bao Q, Loh KP (2012). Graphene photonics, plasmonics, and broadband optoelectronic devices. ACS Nano.

[36] Ghaffari V, Yousefi L (2023). Integrated optical beam steering device using switchable nanoantennas and a reflective metalens. Sci. Rep..

[37] Ra’di Y, Alù A (2018). Reconfigurable metagratings. Acs Photonics.

[38] Dawlaty JM, Shivaraman S, Chandrashekhar M, Rana F, Spencer MG (2008). Measurement of ultrafast carrier dynamics in epitaxial graphene. Appl. Phys. Lett..

[39] Kumar S (2009). Femtosecond carrier dynamics and saturable absorption in graphene suspensions. Appl. Phys. Lett..

[40] Sounas DL, Alù A (2014). Angular-momentum-biased nanorings to realize magnetic-free integrated optical isolation. ACS Photonics.

[41] Qin C, Wang B, Long H, Wang K, Lu P (2016). Nonreciprocal phase shift and mode modulation in dynamic graphene waveguides. J. Lightwave Technol..

[42] Ramaccia D, Sounas DL, Alù A, Bilotti F, Toscano A (2018). Nonreciprocity in antenna radiation induced by space-time varying metamaterial cloaks. IEEE Antennas Wirel. Propag. Lett..

[43] Shi Y, Han S, Fan S (2017). Optical circulation and isolation based on indirect photonic transitions of guided resonance modes. ACS Photonics.

[44] Huang W, Hong J (1992). A transfer matrix approach based on local normal modes for coupled waveguides with periodic perturbations. J. Lightwave Technol..

[45] Huang W, Hong J, Mao Z (1993). Improved coupled-mode formulation based on composite modes for parallel grating-assisted co-directional couplers. IEEE J. Quantum Electron..

[46] Passaro VM, Armenise MN (1995). Analysis of radiation loss in grating-assisted codirectional couplers. IEEE J. Quantum Electron..

[47] Little B, Haus H (1995). A variational coupled-mode theory for periodic waveguides. IEEE J. Quantum Electron..

[48] Sun N-H, Butler JK, Evans GA, Pang L, Congdon P (1997). Analysis of grating-assisted directional couplers using the Floquet-Bloch theory. J. Lightwave Technol..

[49] Taravati S (2018). Giant linear nonreciprocity, zero reflection, and zero band gap in equilibrated space-time-varying media. Phys. Rev. Appl..

[50] Esquius-Morote M, Gómez-Dı JS, Perruisseau-Carrier J (2014). Sinusoidally modulated graphene leaky-wave antenna for electronic beamscanning at THz. IEEE Trans. Terahertz Sci. Technol..

[51] Chen P-Y, Argyropoulos C, Alu A (2012). Terahertz antenna phase shifters using integrally-gated graphene transmission-lines. IEEE Trans. Antennas Propag..

[52] Chen P-Y, Huang H, Akinwande D, Alu A (2014). Graphene-based plasmonic platform for reconfigurable terahertz nanodevices. ACS Photonics.

[53] Nezhad VF, Haddadpour A, Veronis G (2016). Tunable spatial mode converters and optical diodes for graphene parallel plate waveguides. Opt. Express.

[54] Hanson GW (2008). Quasi-transverse electromagnetic modes supported by a graphene parallel-plate waveguide. J. Appl. Phys..

[55] Novoselov KS (2004). Electric field effect in atomically thin carbon films. Science.

[56] Berger C (2004). Ultrathin epitaxial graphite: 2D electron gas properties and a route toward graphene-based nanoelectronics. J. Phys. Chem. B.

[57] Wang F (2008). Gate-variable optical transitions in graphene. Science.

[58] Hendry E, Hale PJ, Moger J, Savchenko A, Mikhailov SA (2010). Coherent nonlinear optical response of graphene. Phys. Rev. Lett..

[59] Hanson GW (2008). Dyadic Green’s functions and guided surface waves for a surface conductivity model of graphene. J. Appl. Phys..

[60] Falkovsky L (2008). Optical properties of grapheme. J. Phys. Conf. Ser..

[61] Correas-Serrano D, Gomez-Diaz J, Sounas D, Alvarez-Melcon A, Alù A (2016). 2016 10th European Conference on Antennas and Propagation (EuCAP).

[62] Falkovsky L, Varlamov A (2007). Space-time dispersion of graphene conductivity. Eur. Phys. J. B.

[63] Ju L (2011). Graphene plasmonics for tunable terahertz metamaterials. Nat. Nanotechnol..

[64] Woessner A (2017). Electrical 2π phase control of infrared light in a 350-nm footprint using graphene plasmons. Nat. Photonics.

[65] Ryzhii V (2012). Effect of plasma resonances on dynamic characteristics of double graphene-layer optical modulator. J. Appl. Phys..

[66] Ryzhii V, Otsuji T, Ryzhii M, Shur MS (2012). Double graphene-layer plasma resonances terahertz detector. J. Phys. D Appl. Phys..

[67] Ryzhii V (2013). Terahertz photomixing using plasma resonances in double-graphene layer structures. J. Appl. Phys..

[68] Slater JC (1958). Interaction of waves in crystals. Rev. Modern Phys..

[69] Brillouin, L.N. Wave propagation in periodic structures: Electric filters and crystal lattices (1953).

[70] Liu M (2011). A graphene-based broadband optical modulator. Nature.

[71] Cullen A (1958). A travelling-wave parametric amplifier. Nature.

[72] Tien P (1958). Parametric amplification and frequency mixing in propagating circuits. J. Appl. Phys..

[73] Kleinert M (2016). Graphene-based electro-absorption modulator integrated in a passive polymer waveguide platform. Opt. Mater. Express.

[74] Yan Z (2011). Growth of bilayer graphene on insulating substrates. ACS Nano.

